# Thermophysical and anion diffusion properties of (U_*x*_,Th_1−*x*_)O_2_

**DOI:** 10.1098/rspa.2014.0427

**Published:** 2014-11-08

**Authors:** Michael W. D. Cooper, Samuel T. Murphy, Paul C. M. Fossati, Michael J. D. Rushton, Robin W. Grimes

**Affiliations:** Department of Materials, Imperial College, London SW7 2AZ, UK

**Keywords:** nuclear fuel, uranium dioxide, thermal expansion, bulk modulus, specific heat, anion diffusion

## Abstract

Using molecular dynamics, the thermophysical properties of the (U_*x*_,Th_1−*x*_)O_2_ system have been investigated between 300 and 3600 K. The thermal dependence of lattice parameter, linear thermal expansion coefficient, enthalpy and specific heat at constant pressure is explained in terms of defect formation and diffusivity on the oxygen sublattice. Vegard's law is approximately observed for solid solution thermal expansion below 2000 K. Different deviations from Vegard's law above this temperature occur owing to the different temperatures at which the solid solutions undergo the superionic transition (2500–3300 K). Similarly, a spike in the specific heat, associated with the superionic transition, occurs at lower temperatures in solid solutions that have a high U content. Correspondingly, oxygen diffusivity is higher in pure UO_2_ than in pure ThO_2_. Furthermore, at temperatures below the superionic transition, oxygen mobility is notably higher in solid solutions than in the end members. Enhanced diffusivity is promoted by lower oxygen-defect enthalpies in (U_*x*_,Th_1−*x*_)O_2_ solid solutions. Unlike in UO_2_ and ThO_2_, there is considerable variety of oxygen vacancy and oxygen interstitial sites in solid solutions generating a wide range of property values. Trends in the defect enthalpies are discussed in terms of composition and the lattice parameter of (U_*x*_,Th_1−*x*_)O_2_.

## 1. Introduction

UO_2_ has been studied extensively as the main component of conventional nuclear fuel. It is also blended with other actinide oxides, such as ThO_2_ [1] and PuO_2_ [2,3], forming mixed oxide (MOX) fuel. Alternatively, long-lived minor actinides can be separated from nuclear waste and blended with UO_2_ or MOX for transmutation in a reactor- [4] or accelerator-driven system [5,6]. Owing to its relative abundance, thorium is considered as an important candidate for MOX, whereby Th^232^ transmutates in reactor to U^233^, which can then undergo fission. As such, it is important to understand the underlying mechanisms that govern the thermophysical and diffusion properties in mixed oxides, owing to a non-uniform cation sublattice.

For UO_2_, there is a deviation from linear thermal expansion and a classical Debye description of the constant pressure specific heat above 1300 K [7–16]. At 2670 K (0.85T_*m*_), there is a peak in specific heat owing to a pre-melting transition or superionic transition as seen in other fluorite structures [7]. Below the transition, it is not yet clear to what extent the excess specific heat and thermal expansion is driven by oxygen disorder versus electronic-defect contributions, or over what temperature ranges these effects may dominate.

A great deal of work has been carried out using atomistic simulation to study candidate actinide oxide components of nuclear fuel [17–21]. The ability of an interatomic potential to accurately reproduce the thermophysical properties of UO_2_, such as lattice parameter, elastic constants, thermal conductivity and specific heat over a wide range of temperatures, has often been used as a key discriminator for the suitability of a potential set [17–20]. Similarly, Potashnikov *et al.* [22] compared the ability of a number of interatomic potentials to predict oxygen diffusivity in UO_2_ using molecular dynamics (MD). However, experimental data for oxygen diffusion will be influenced by the presence of point defects arising owing to materials processing conditions, and it not necessarily comparable with the perfect crystal calculations of Potashnikov [22]. For example, the enhancement of oxygen diffusivity owing to non-stoichiometry in UO_2_ has been demonstrated recently by Govers *et al.* [23] and shown experimentally by Belle [24]. Similarly, enhanced diffusivity owing to Schottky defects was also identified by Potashnikov *et al.* [22]. Therefore, it is not surprising that many simulations on perfect crystals predict lower oxygen diffusivity than experiment.

Recently, a potential set has been derived that accurately reproduces a wide range of thermomechanical and thermophysical properties for AmO_2_, CeO_2_, CmO_2_, NpO_2_, PuO_2_, ThO_2_ and UO_2_, between 300 and 3000 K [25]. In particular, this potential accurately represents the individual elastic constants of the actinide oxides and reproduces the Cauchy violation (C_12_≠C_44_) by introducing many-body interactions using the embedded atom method (EAM) [26] without the necessity for the shell model [27]. As a result, a significant improvement in the ability of empirical interatomic potentials to reproduce the bulk modulus over a large range of temperatures has been achieved. Importantly, this potential set employs the same description of oxygen–oxygen interactions throughout, enabling the simulation of actinide oxide solid solutions. Furthermore, it accurately reproduces the melting points of UO_2_ and ThO_2_ well, making it particularly suitable for investigating (U_*x*_,Th_1−*x*_)O_2_ solid solutions.

Here, we investigate, using atomistic simulation, the lattice parameter, linear coefficient of thermal expansion, enthalpy and specific heat at constant pressure for (U_*x*_,Th_1−*x*_)O_2_ between 300 and 3600 K for *x* = 0.00, 0.25, 0.50, 0.75 1.00. Furthermore, the influence of solid solution composition on oxygen-defect formation, oxygen diffusivity and the superionic transition is reported.

## 2. Methodology

MD simulations are carried out using LAMMPS [28], and the set of interatomic potentials derived previously [25].^1^ The model combines a pair potential description of each system with the many-body EAM description of Daw & Baskes [26]. As such, the potential energy, *E*_*i*_, of an atom *i* with respect to all other atoms can be written as

2.1 $${\mathrm{E3}}_{\text{i}}=\frac{1}{2}\sum_{\text{j}}\phi_{\alpha \beta }(r_{ij})-G_{\alpha }\sqrt{\sum_{\text{j}}\sigma_{\beta }(r_{\text{ij}})},$$

where the pairwise interaction between two atoms *i* and *j*, separated by *r*_*ij*_, is given by *ϕ*_*αβ*_(*r*_*ij*_). This has both long range electrostatic, *ϕ*_*C*_(*r*_*ij*_), and short range contributions. Coulombic interactions are calculated using the Ewald method [31] with the particle–particle particle–mesh (PPPM) implementation of the method being adopted within MD calculations in order to improve computational efficiency [32]. The short range contributions are described using Morse, *ϕ*_*M*_(*r*_*ij*_), and Buckingham, *ϕ*_*B*_(*r*_*ij*_), potential forms, as given by equation (2.2) [33,34]. *α* and *β* are used to label the species of atom *i* and atom *j*, respectively,

2.2 $$\begin{array}{r} \phi_{\alpha \beta }(r_{\text{ij}})=\phi_{C}(r_{\text{ij}})+\phi_{B}(r_{\text{ij}})+\phi_{M}(r_{\text{ij}}), \end{array}$$

2.3 $$\begin{array}{r} \phi_{C}(r_{\text{ij}})=\frac{q_{\alpha }q_{\beta }}{4\pi \epsilon_{0}r_{\text{ij}}}, \end{array}$$

2.4 $$\begin{array}{r} \phi_{B}(r_{\text{ij}})=A_{\alpha \beta }\exp\left(\frac{-r_{\text{ij}}}{\rho_{\alpha \beta }}\right)-\frac{C_{\alpha \beta }}{r_{\text{ij}}^{6}} \end{array}$$

2.5 $$\begin{array}{r} \text{and}\;\phi_{M}(r_{\text{ij}})=D_{\alpha \beta }[\exp(-2\gamma_{\alpha \beta }(r_{\text{ij}}-r_{0}))-2\exp(-\gamma_{\alpha \beta }(r_{\text{ij}}-r_{0}))], \end{array}$$

where *A*_*αβ*_, *ρ*_*αβ*_, *C*_*αβ*_, *D*_*αβ*_, *γ*_*αβ*_ and *r*_0_ are empirical parameters that describe the pair interactions between atom *i* and atom *j*. These have been reported previously for AmO_2_, CeO_2_, CmO_2_, NpO_2_, PuO_2_, ThO_2_ and UO_2_ [25]. For the study of solid solutions, further mixed cation–cation pair interactions (e.g. *ϕ*_U−Th_) must also be defined. As was the case for the self cation–cation pair interactions [25], the mixed cation–cation interactions are dominated by Coulombic interactions at the separations exhibited by the fluorite structure. Hence, it is not possible to fit these parameters to experimental bulk properties. Instead, the assumptions made previously for self-interactions are extended here to mixed cation–cation pair potentials. The description of covalency predicted by the Morse potential is not required for cation–cation pairs and is therefore excluded for these interactions (e.g. *D*_U−Th_=0 *eV*). The pre-exponential term of the Buckingham potential is the same for all cation–cation pairs and is based on the parameter reported by Grimes & Catlow [35] (e.g. *A*_U−Th_=18600 *eV*). However, the reported self cation–cation *ρ*_*αα*_ parameters are scaled to cation radius [25]. Equation (2.6) [36] is used to determine *ρ*_*αβ*_ parameters for mixed cation–cation pairs whose values are reported in table 1,

2.6 $$\rho_{\alpha \beta }=\sqrt{\rho_{\alpha \alpha }.\rho_{\beta \beta }}.$$

**Table 1.** *ρ*_*αβ*_ parameters for the pairwise interactions of mixed cation–cation pairs determined using equation (2.6). As the table is symmetric (i.e. *ρ*_*αβ*_=*ρ*_*βα*_), values are only reported once.

(Å)	Ce	Th	U	Np	Pu	Am	Cm
Ce	0.2664	0.2772	0.2705	0.2678	0.2651	0.2637	0.2637
Th	—	0.2884	0.2815	0.2786	0.2758	0.2743	0.2743
U	—	—	0.2747	0.2719	0.2691	0.2677	0.2677
Np	—	—	—	0.2692	0.2664	0.2650	0.2650
Pu	—	—	—	—	0.2637	0.2623	0.2623
Am	—	—	—	—	—	0.2609	0.2609
Cm	—	—	—	—	—	—	0.2609

The second term in equation (2.1) uses the EAM to introduce a many-body perturbation to the more dominant pairwise interactions. The derivation of the parameters and a description of the functional terms used in the EAM component are given in reference [25].

The thermal expansion, specific heat and oxygen diffusivity in the (U_*x*_,Th_1−*x*_)O_2_ system are investigated for the UO_2_, (U_0.75_,Th_0.25_)O_2_, (U_0.5_,Th_0.5_)O_2_, (U_0.25_,Th_0.75_)O_2_ and ThO_2_ compositions. Solid solution crystal structures are created by randomly distributing U^4+^ and Th^4+^ cations on the 4a Wyckoff sites (fluorite actinide sites) throughout a supercell of 10×10×10 fluorite unit cells. These structures are equilibrated for 40 ps for temperatures between 300 and 3600 K at 25 K intervals with the thermophysical properties (lattice parameter and enthalpy) obtained from averages taken over the final 2 ps of the simulation. A 2 fs timestep is used in the NPT ensemble with Nosé–Hoover thermostat and barostat times of 0.1 and 0.5 ps, respectively. For each composition, this is repeated for 10 randomly generated structures.

The equilibrated solid solution structures are also used to determine oxygen diffusivity. The oxygen mean-squared displacement (MSD) is calculated in the NVE ensemble for 1 ns with a 1 fs timestep for a range of temperatures from 2000 to 3600 K at 100 K intervals. From this the diffusivity, *D*, is calculated using the following equation [37],

2.7 $$D=\frac{\left\langle R_{O}^{2}\right\rangle }{6t},$$

where $\left\langle R_{O}^{2}\right\rangle$ is the total oxygen MSD and *t* is the simulation time.

The point defects present in a simulation box are counted by analysing structural information extracted from the configuration. The following procedure is carried out independently on the two sublattices present (oxygen and actinide). The average distribution of atoms around a site of the sublattice *A* is calculated. This is a three-dimensional equivalent of the partial radial distribution function:

2.8 $$\mu_{A}(r)=\frac{1}{N_{A}\;dV(r)}\sum_{i}^{N_{A}}\sum_{j}^{N_{A}}\delta (r_{ij}-r),$$

where *μ*_*A*_(**r**) is proportional to the probability of finding an atom of sublattice *A* in a volume *dV* around a position **r** from a lattice site; *i* and *j* are two atoms of sublattice *A* and *N*_*A*_ is the number of atoms on this sublattice. The local maxima of this scalar field indicate the positions most likely to be occupied by a neighbour. When all the sites on a sublattice have the same local symmetry, these are the positions of the neighbours {**R**_*A*,*λ*_}, with *λ*∈{1,…,*Z*^*A*^}, *Z*^*A*^ being the connectivity of the sublattice (number of neighbours for each site). For the fluorite structure, *Z* is 6 for the oxygen sublattice (simple cubic), and 12 for the actinide sublattice (face centred cubic). The local maxima of *μ*_*A*_(**r**) have a width that can be estimated from the full width at half maximum (FWHM). This is related to the fluctuations of the neighbour positions caused by thermal oscillations around the lattice sites. Thus, one can define a distance criterion *δ*_*A*_ based on the FWHM, so that an atom *j* is said to occupy the neighbour site *λ* around the atom *i*, if

2.9 $$\left|r_{ij}-R_{A,\lambda }\right|<\delta_{A}.$$

The connectivity, *Z*_*i*_, of the atom *i* is then the number of such valid neighbours.

Having calculated *Z* for each atom, those that have the same connectivity as their sublattice are in a perfect environment and ignored for the rest of the defect counting procedure. The vacancies are detected using the fact that several atoms would have a common missing neighbour. A virtual atom, not present in the real configuration, is added to such positions and treated as a regular atom during the remaining defect detection. The last step is repeated until no valid vacancy position is found. At this point, the atoms that have a low connectivity are either part of extended defects, which are ignored in this study, or interstitial atoms. The interstitials are characterized by a connectivity number lower than 3.

This procedure gives the number of vacancy and interstitial defects. When carried out on successive snapshots of constant-temperature MD simulations, the concentration of defects can be estimated as a function of temperature. In this work, snapshots are taken at 1 ps intervals for simulations lasting 25 ps equilibrated at temperatures ranging from 2300 to 3200 K every 100 K. The defect concentrations are then averaged over all snapshots for a given composition to obtain the thermally equilibrated defect concentrations in each solid solution.

For the energy minimization calculations of isolated oxygen vacancy and interstitial defect enthalpies, the same supercells described above are employed. Given the complex structures of these solid solutions, the perfect supercells are subjected to a rigorous energy minimization procedure to ensure they are fully relaxed. This consists of an initial minimization under constant volume conditions using a damped dynamics algorithm [38] followed by a constant pressure step using a conjugate gradient method before a final optimization step employing a steepest descent procedure with fixed lattice parameters. Once the solid solution supercells are fully optimized, point defects were introduced into the simulation supercells by either removing (vacancy) or adding (interstitial) oxygen atoms into the supercell. The defective supercells are energy minimized with the lattice parameters fixed in order to represent the dilute limit with the defect enthalpy, *dE*, calculated using

2.10 $$dH=H_{\mathrm{defect}}-H_{\mathrm{perfect}},$$

where *H*_perfect_ and *H*_defect_ are the total enthalpies of the perfect and defective cells, respectively. The oxygen Frenkel enthalpies for UO_2_ and ThO_2_ are converged to within 0.1 eV for the 10×10×10 supercell compared with the fully isolated enthalpies given previously [25]; therefore, the defect enthalpies are considered to be converged with respect to system size.

For the oxygen vacancy simulations, an oxygen is removed from each of the oxygen lattice sites, in all of the 10 simulation supercells for each composition, resulting in a total of 80 000 defect simulations. Similarly, the oxygen interstitial defect enthalpy is calculated at every possible interstitial site, in all of the supercells, leading to a total of 40 000 defect simulations. This large number of simulations allows us to access the statistical distribution of the defect enthalpies arising from the random arrangement of cations on the 4a Wyckoff sites. As this approach generates a very large number of unique defect energies, the dataset has been grouped into bins of width 0.01 eV for ease of manipulation and to enable useful presentation of these results.

## 3. Results and discussion

### (a) Oxygen diffusivity

By assuming an Arrhenius relationship, equation (3.1), *D* is plotted logarithmically as a function of 1/*T*, so that, the gradient is proportional to the activation enthalpy, *H*_*a*_.

**Figure 1.**
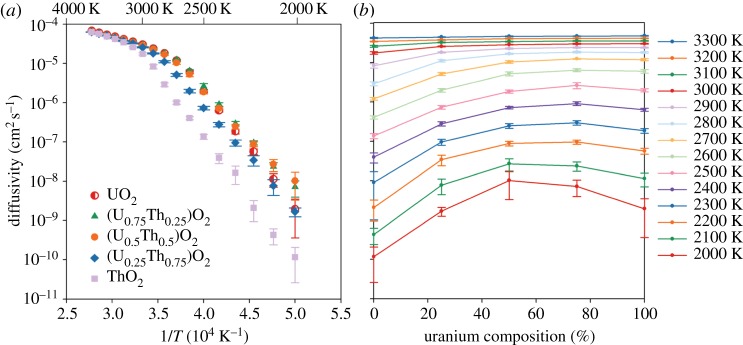
Oxygen diffusivity as function of (*a*) temperature and (*b*) composition for UO_2_, ThO_2_ and three compositions of the (U_*x*_,Th_1−*x*_)O_2_ solid solution. Values are given by the average from 10 different random structures and errors bars represent the standard deviation. (Online version in colour.)

**Figure 2.**
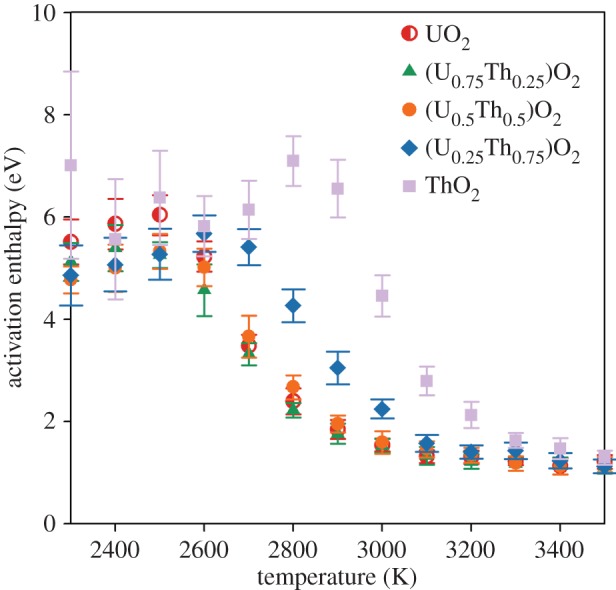
The activation enthalpy, *H*_*a*_, for oxygen migration as function of temperature for UO_2_, ThO_2_ and three compositions of the (U_*x*_,Th_1−*x*_)O_2_ solid solution. The variation in activation enthalpy with temperature indicates non-Arrhenius diffusion mostly owing to the superionic transition. Values are given by the average from 10 different random structures and errors bars represent the standard deviation. (Online version in colour.)

3.1 $$D=D_{0}\exp\left(-\frac{H_{a}}{k_{B}T}\right),$$

where *D*_0_ is the pre-exponential term, *k*_B_ is the Boltzmann constant and *T* is temperature. For each composition, *D* is averaged over all 10 randomly generated structures and plotted in figure 1*a*; error bars indicate the standard deviation. Regions of constant gradient, and thus activation enthalpy, indicate temperature regimes with a common diffusion mechanism. As in the study of Potashnikov *et al.* [22], figure 1*a* shows that the transition between the fully crystalline low temperature and superionic high temperature behaviour occurs over a range of temperature specific to each composition. Similarly, figure 2 highlights the change in activation enthalpy during the transition. It can be seen that the transition occurs at a higher temperature in ThO_2_ compared with UO_2_. However, the addition of thorium to form (U_0.75_,Th_0.25_)O_2_ and (U_0.5_,Th_0.5_)O_2_ solid solutions does not appear to significantly increase the superionic transition temperature (and may even have reduced it for (U_0.75_,Th_0.25_)O_2_). It is not until the (U_0.25_,Th_0.75_)O_2_ solid solution that the thorium content is significant enough to increase the superionic phase transition temperature. In table 2, the approximate range of superionic transition temperatures has been summarized. The significance of this for the high temperature thermophysical properties is discussed in §3*d*,*e*.

**Table 2.** Based on the range of temperatures over which the activation energy (figure 2) and, thus, mechanism for oxygen diffusion changes the lower and upper temperatures of superionic transition have been summarized for each solid solution composition.

composition, *x*	lower (K)	upper (K)
1, 0.75 and 0.5	2500	2900
0.25	2700	3100
0	2900	3300

Figure 1*b* shows the oxygen diffusivity as a function of uranium composition for a range of temperatures. There is a clear enhancement of oxygen diffusivity below the superionic transition temperature for the solid solutions compared with pure UO_2_ and ThO_2_. From about 2200 to 2700 K, oxygen diffusivity is greatest in (U_0.75_,Th_0.25_)O_2_. For 2000 and 2100 K, (U_0.5_,Th_0.5_)O_2_ exhibits the highest oxygen diffusivity and (U_0.25_,Th_0.75_)O_2_ is approaching the same level as pure UO_2_. Furthermore, if the trend for (U_0.25_,Th_0.75_)O_2_ in figure 1 continues below 2000 K, it is possible that the oxygen diffusivity may also exceed that of UO_2_; however, further diffusivity calculations at lower temperatures must be carried out in order to confirm this prediction. The role of such solid solutions in enhancing oxygen diffusivity in mixed oxide fuels is therefore expected to be significant at reactor operating temperatures and may impact the release of fission products that occupy and migrate via the oxygen sublattice (e.g. I^−^) [35].

### (b) Oxygen-defect concentrations

**Figure 3.**
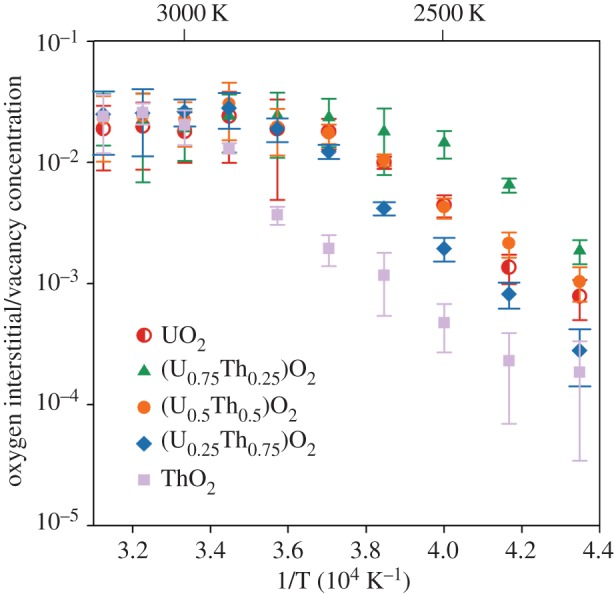
The oxygen interstitial and vacancy concentration ([O′′_*i*_]=[V${\;}_{O}^{∙∙}]$) as a function of temperature for UO_2_, ThO_2_ and three compositions of the (U_*x*_,Th_1−*x*_)O_2_ solid solution. The number of interstitial–vacancy pairs is normalized against the total number of oxygen ions in the system to get the defect concentration. (Online version in colour.)

Oxygen-defect concentrations underpin the thermophysical properties of actinide oxides as well as the transition to superionicity. It is, therefore, important to calculate the defect concentrations that are generated during our high temperature simulations in order to correctly identify which defects are responsible for the high temperature phenomena. The defect concentrations are calculated using a method developed for the purpose of this study. Figure 3 shows the oxygen interstitial concentration (fraction of oxygen ions off their lattice sites) plotted logarithmically as a function of 1/*T*, such that the gradient is proportional to the defect formation enthalpy for an interstitial–vacancy pair. Below the superionic transition, the defect populations were dominated by tightly bound pairs of oxygen vacancies and interstitials, such that $\left[V_{O}^{∙∙}]=[O_{i}^{\prime \prime }\right]$. As demonstrated in previous work using energy minimization [25], the recombination of first nearest neighbour oxygen vacancy–interstitial pairs with this potential set is spontaneous (i.e. no barrier). Although the oxygen ion can exist off its perfect lattice site for a finite amount of time, it is not at a stable defect site and must be distinguished from stable Frenkel pairs that exist beyond first nearest neighbour positions by being labelled pseudo-Frenkel defects, a name used previously for studies on similar systems [39–41]. The oxygen pseudo-Frenkel enthalpies of 4.42 eV for UO_2_ and 4.70 eV for ThO_2_, corresponding to the gradients of figure 3, are therefore not equivalent to those reported previously [25] that were calculated for stable Frenkel pairs (which have a greater vacancy–interstitial separation). This spontaneous recombination of first-neighbour pairs on the oxygen sublattice has also been observed in UO_2_ using other empirical potentials [39,40] and in other similar structures, such as pyrochlores [41]. Figure 3 shows that at higher temperatures the fraction of oxygen anions in these pseudo-Frenkel positions increases. As a consequence, there is an increase in system enthalpy and lattice parameter associated with the pseudo-defect enthalpies and psuedo-defect volumes, respectively (see §3*d*,*e*). As the oxygen disorder increases during the superionic transition (table 2), the defect counting method can no longer identify a clear oxygen sublattice and, as a result, fails to identify the correct number of interstitials. This point is characterized by a sharp decrease in the gradient of figure 3 that helps identify the superionic transition, showing that the transition temperature is lower in UO_2_ than ThO_2_ and potentially even lower in (U_0.75_,Th_0.25_)O_2_.

### (c) Oxygen point-defect enthalpies

To study the possibility of enhanced permanent oxygen-defect concentrations in the solid solutions, it is necessary to identify the point defect formation enthalpies. Unlike the pure end member systems oxygen sites in a solid solution are different and consequently, there is a wide range of defect enthalpies owing to the various environments surrounding the vacancy. A similar observation has been made for the As vacancy in In_*x*_Ga_1−*x*_As [42].

**Figure 4.**
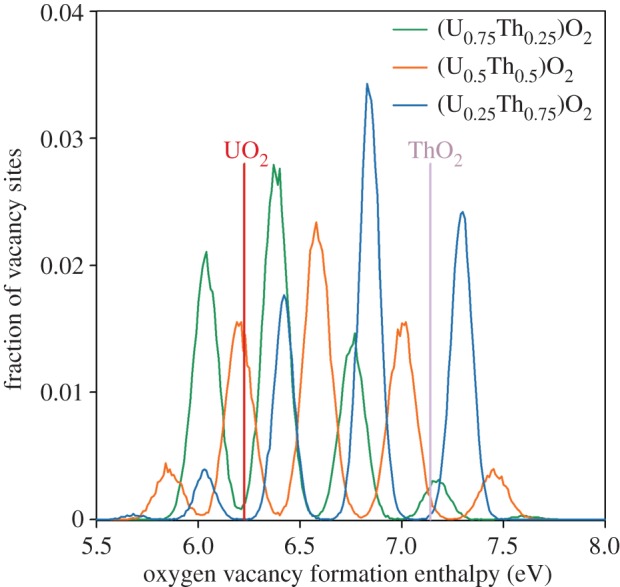
The fraction of oxygen sites that lie within 0.005 eV of the corresponding oxygen vacancy formation enthalpy for UO_2_, ThO_2_ and three compositions of the (U_*x*_,Th_1−*x*_)O_2_ solid solution. From left to right, the peaks correspond to sites coordinated by 4, 3, 2, 1 and 0 uranium ions (or 0, 1, 2, 3 and 4 thorium ions). There is only one value for UO_2_ and ThO_2_ as represented by vertical lines. (Online version in colour.)

**Figure 5.**
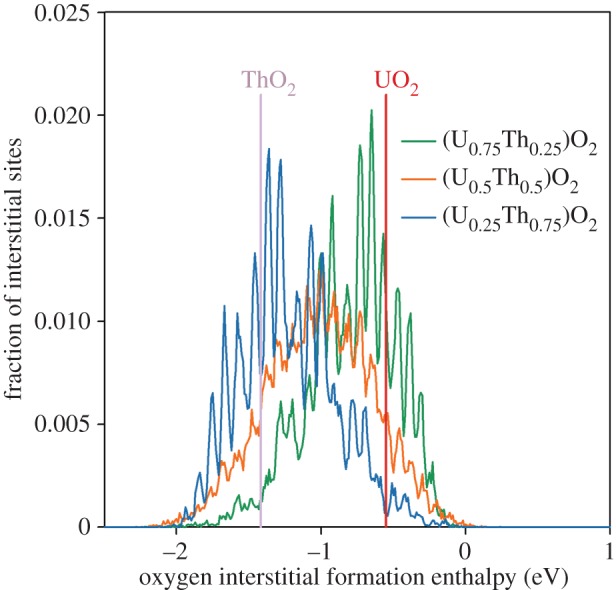
The fraction of oxygen interstitial sites that lie within 0.005 eV of the corresponding oxygen interstitial formation enthalpy for UO_2_, ThO_2_ and three compositions of the (U_*x*_,Th_1−*x*_)O_2_ solid solution. Spikes in the data correspond to particular configurations of cations on the six first nearest neighbour cation sites. Note that the (U_0.75_,Th_0.25_)O_2_ and (U_0.25_,Th_0.75_)O_2_ distributions are symmetrical about the centre of the (U_0.5_,Th_0.5_)O_2_ peak. There is only one value for UO_2_ and ThO_2_ as represented by vertical lines. (Online version in colour.)

Figure 4 identifies the fraction of oxygen sites that lie within 0.005 eV of a given oxygen vacancy formation enthalpy. This shows that for each composition there are five peaks that correspond to the five different first nearest cation neighbour coordination of the oxygen site, with the lowest and highest enthalpy peaks coordinated by 4 and 0 uranium ions, respectively. The skew in peak heights corresponds to the solid solution composition. For example, the proportion of sites fully coordinated by uranium ions (lowest enthalpy peak) is greatest in (U_0.75_,Th_0.25_)O_2_. Additionally, there is a shift in the peaks owing to lattice parameter, whereby, all oxygen vacancy enthalpies are shifted down for solid solutions with a greater lattice parameter (i.e. higher thorium content; see §3*d*). Therefore, the peak corresponding to fully uranium-coordinated sites is always lower for solid solutions compared with the pure UO_2_ system (shown by the vertical red line). The lattice parameter effect is most clear for the (U_0.25_,Th_0.75_)O_2_ system despite this peak being small. Similarly, oxygen interstitial enthalpies are shifted down for solid solutions with a larger lattice parameter, as illustrated in figure 5.

At lower temperatures, the oxygen vacancies with higher formation enthalpies play a proportionately lesser role in oxygen transport than at higher temperatures. This is demonstrated by enhanced diffusivity in the solid solutions that exhibit a greater proportion of oxygen defects with lower formation enthalpies (figure 1*b*). This is seen clearly for (U_0.75_,Th_0.25_)O_2_ and (U_0.5_,Th_0.5_)O_2_, which both have a significant number of vacancy formation enthalpies below that exhibited by UO_2_. The prediction in §3*a* that oxygen diffusivity in (U_0.25_,Th_0.75_)O_2_ may also exceed diffusion in UO_2_ below 2000 K can now be understood as a consequence of it containing the lowest enthalpy peak in figure 4. For the full oxygen Frenkel enthalpy, oxygen interstitials must also be included. Figure 5 further supports enhanced oxygen disorder as all solid solution compositions exhibit a significant number of interstitials with lower formation enthalpies than for the end members.

### (d) Thermal expansion

**Figure 6.**
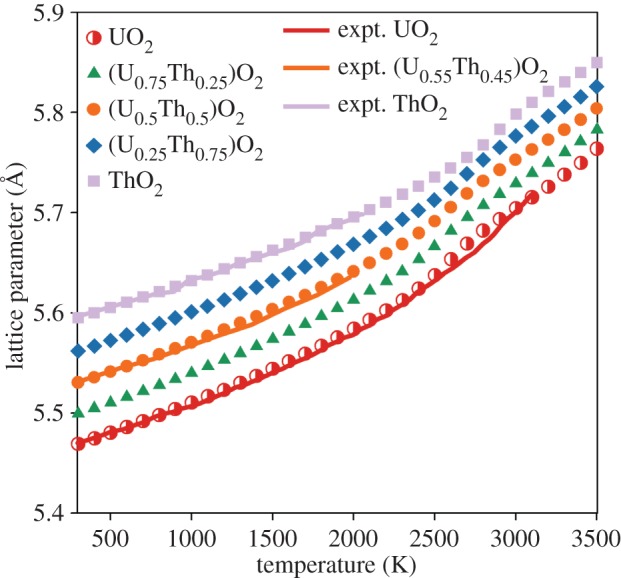
The variation of lattice parameter as a function of temperature is shown for UO_2_, ThO_2_ and three compositions of the (U_*x*_,Th_1−*x*_)O_2_ solid solution. Values are given by the average from 10 different random structures. The errors bars from the standard deviation are too small to see. Experimental data for UO_2_ [16], ThO_2_ [43] and (U_0.55_,Th_0.45_)O_2_ [44] have been included showing good agreement with the model. (Online version in colour.)

Figure 6 shows the increase in the lattice parameter as a function temperature for a given composition averaged over the 10 randomly generated 10×10×10 structures. Experimental data for UO_2_ [16], ThO_2_ [43] and (U_0.55_Th_0.45_)O_2_ [44] are also included and show very good agreement with the predictions of the potential. Figure 6 illustrates a significant increase (or ‘bump’) in thermal expansion for all compositions of solid solution as well as for the pure systems at high temperature (2300–3300 K), below this temperature, Vegard's law [45] is obeyed. The ‘bump’ in lattice parameter can first be attributed to the defect volumes associated with the formation of a large number of oxygen defects and after that by the volume change owing to the superionic transition. This is more clearly demonstrated by using the first derivative of the lattice parameter with respect to temperature to calculate the linear thermal expansion coefficient, see equation (3.2).

**Figure 7.**
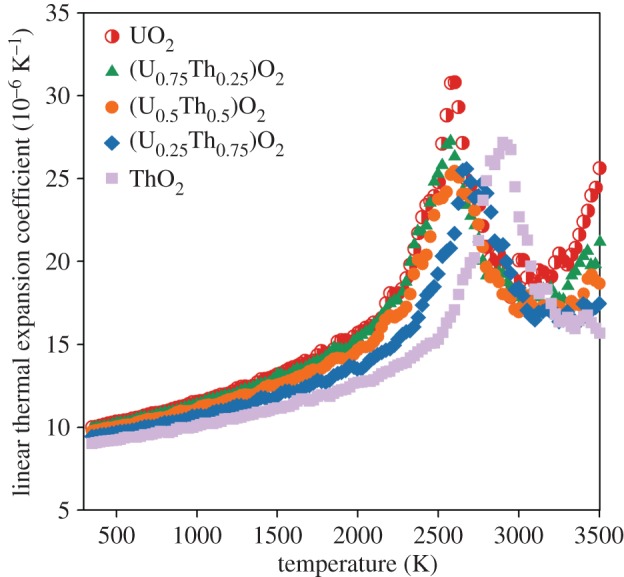
The linear thermal expansion coefficient as a function of temperature is shown for UO_2_, ThO_2_ and three compositions of the (U_*x*_,Th_1−*x*_)O_2_ solid solution. Values are given by the average from 10 different random structures. The errors bars from the standard deviation are too small to see. (Online version in colour.)

3.2 $$\alpha_{P}(L)=\frac{1}{L}{\left(\frac{\partial L}{\partial T}\right)}_{P},$$

where the first derivative of lattice parameter, (∂*L*/∂*T*)_*P*_, is calculated by fitting a straight line to the lattice parameter at a given temperature and the data points at ±25 K either side. Figure 7 shows the variation of linear thermal expansion coefficient as function of temperature. The peak in linear thermal expansion coefficient corresponds closely to the range of temperatures for the superionic transition for each composition (table 2). For UO_2_, (U_0.75_Th_0.25_)O_2_ and (U_0.5_Th_0.5_)O_2_, the peak is at around 2600 K, in close agreement with the experimental value for the superionic transition temperature of 2670 K for UO_2_ [7]. For (U_0.25_Th_0.75_)O_2_ and ThO_2_, the peak is at approximately 2700 and 2950 K, respectively. For UO_2_, (U_0.75_Th_0.25_)O_2_ and (U_0.5_Th_0.5_)O_2_ a second very high temperature peak is identified that is associated with the creation of cation defects; however, as these peaks are above the UO_2_ melting point predicted by this potential [25], it is outside the regime of interest for this study.

### (e) Enthalpy and specific heat

**Figure 8.**
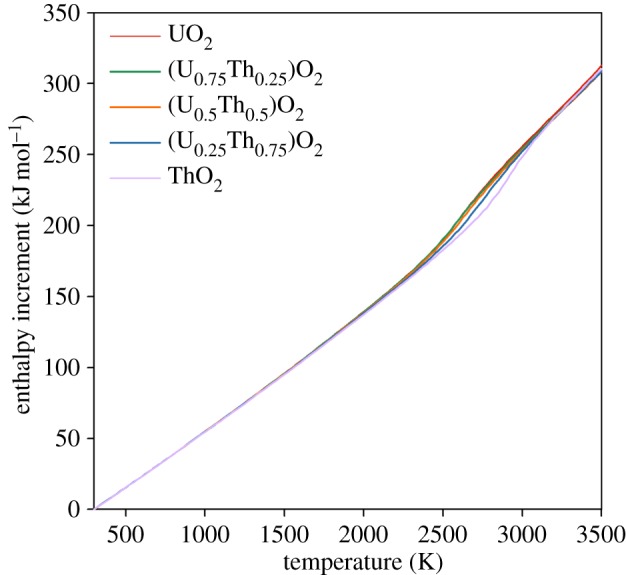
The change in enthalpy as function of temperature relative to the enthalpy at 300 K. This is shown for UO_2_, ThO_2_ and three compositions of the (U_*x*_,Th_1−*x*_)O_2_ solid solution. Values are given by the average from 10 different random structures. The errors bars from the standard deviation are too small to see. (Online version in colour.)

**Figure 9.**
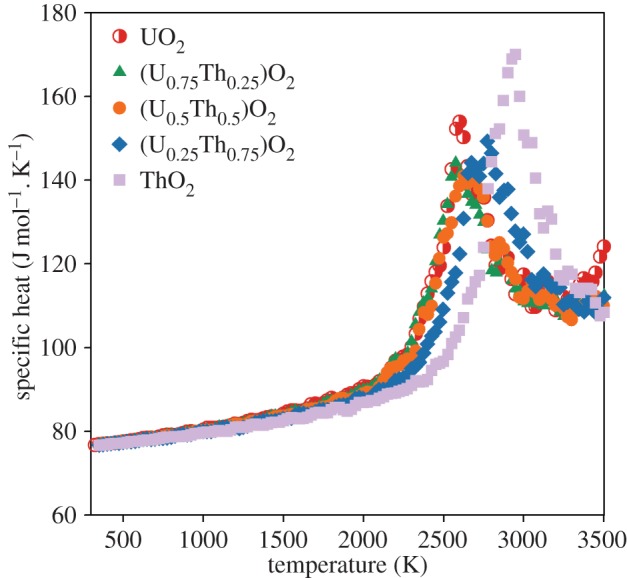
The constant pressure-specific heat capacity as function of temperature relative to the enthalpy at 300 K for UO_2_, ThO_2_ and three compositions of the (U_*x*_,Th_1−*x*_)O_2_ solid solution. Values are given by the average from 10 different random structures. The errors bars from the standard deviation are too small to see. (Online version in colour.)

In addition to the volume change owing to oxygen-defect formation and the superionic phase transition, there is an associated change in system enthalpy. Figure 8 shows the enthalpy increment (increase in enthalpy with respect to standard conditions) as a function of temperature (i.e. H(T)–H(298 K)) averaged over the 10 randomly generated structures for each solid solution composition. The enthalpy increment increases approximately linearly with temperature below 1500 K. Between 2300 and 3300 K, the enthalpy increment as a function of temperature increases more significantly. The first derivative of the enthalpy increment with respect to temperature is used to calculate the specific heat capacity at constant pressure using the following relationship,

3.3 $$c_{p}=\frac{1}{n}{\left(\frac{\partial H}{\partial T}\right)}_{P},$$

where *n* is the number of moles and the first derivative of enthalpy, (∂*H*/∂*T*)_*P*_, is calculated by fitting a straight line to the enthalpy at a given temperature and the data points at ±25 K either side. Figure 9 indicates a gradual increase in the specific heat until around 2000 K at which point the specific heat increases more rapidly due the enthalpy required to create oxygen disorder. The peak in specific heat is commensurate with the superionic transition (table 2), which occurs close to the same temperature as the peak in the linear thermal expansion coefficient for each composition.

Despite the omission of electronic defects from these empirical calculations, figures 6–9 show that anion disorder contributes significantly to excess thermal expansion and specific heat capacity, while also indicating the peaks are commensurate with the superionic transition. However, comparison of our results with the experimental data for UO_2_ specific heat capacity [16] indicates that oxygen disorder is not sufficient to account for excess specific heat at intermediate temperatures which may be accounted for by electronic defects [12].

## 4. Conclusions

Using MD, the superionic transition in (U_*x*_,Th_1−*x*_)O_2_ is investigated for compositions where *x* equals 0.00, 0.25, 0.50, 0.75 and 1.00. This is identified by the change in activation enthalpy, and thus diffusion mechanism, for oxygen migration. It is shown that reduced oxygen-defect enthalpies in the three solid solution compositions studied here contribute to enhanced oxygen diffusivity below the superionic transition.

The creation of oxygen pseudo-Frenkel pairs and subsequently the superionic transition causes a ‘bump’ in the lattice parameter, thermal expansion coefficient, enthalpy and specific heat capacity for all (U_*x*_,Th_1−*x*_)O_2_ compositions (including end members). The onset of the superionic transition was calculated to occur at similar temperatures for UO_2_, (U_0.75_,Th_0.25_)O_2_ and (U_0.5_,Th_0.5_)O_2_, although it appears to occur slightly earlier in (U_0.75_,Th_0.25_)O_2_ when the oxygen activation enthalpy is considered. The superionic transition temperatures for (U_0.25_,Th_0.75_)O_2_ and ThO_2_ are higher. The change in volume owing to the creation of oxygen disorder explains the high temperature lattice expansion, whereas the latent heat required to undergo the superionic transition is responsible for a peak in the specific heat.

The enhanced low temperature-defect formation and anion diffusion in high U content solid solutions, compared with the UO_2_ end member, has implications for the mobility of fission products, such as iodine, which may be transported via the oxygen sublattice [35].

## Footnotes

1 Supplementary material describing the use of this potential for use in GULP [29], LAMMPS [28] and DL-POLY [30] is provided at http://abulafia.mt.ic.ac.uk/potentials/actinides.
